# First insight of the genome-wide association study and genomic prediction into enteritis disease (*Vibrio harveyi*) resistance trait in the lined seahorse (*Hippocampus erectus*)

**DOI:** 10.3389/fimmu.2024.1474746

**Published:** 2024-10-03

**Authors:** Siping Li, Xin Liu, Fengyuan Shen, Tingting Lin, Dong Zhang

**Affiliations:** ^1^ Key Laboratory of Inland Saline-alkaline Aquaculture, Ministry of Agriculture and Rural Affairs, Shanghai, China; ^2^ East China Sea Fisheries Research Institute, Chinese Academy of Fishery Sciences, Shanghai, China

**Keywords:** the lined seahorse, enteritis disease resistance, GWAS, SNP, genomic selection

## Abstract

Enteritis caused by *Vibrio* is a highly die-off disease that severely impeded substantial production in seahorse aquaculture. In the present study, challenged with LD50 of concentration of Vibrio harveyi, a total of 161 of susceptible and 166 of resistant individuals were allocated into binary survival phenotypes, thus, to firstly investigate the genetic architecture by genome-wide association study (GWAS) analysis, as well as to evaluate the feasibility of genomic selection (GS) in enteritis disease resistance trait of the lined seahorse *Hippocampus erectus*. Results indicated that the heritability for resistance to *Vibrio harveyi* was estimated to be 0.10. And a set of 10 significant/suggestive SNPs in a multiple chromosomes localization were identified, explaining 7.76% to 13.28% of genetic variance. Associated 82 of candidate genes were clustered into signal transduction, cell proliferation, response of external stress, bacteria defence, and anti-inflammatory processes. Moreover, the potential performance of genomic selection (GS) in application in selective breeding for enteritis disease resistance seahorses was assessed by genomic prediction (GP). In general, the predictive accuracy of the genomic estimated breeding value (GEBV) of BayesC exceeded the rrBLUP, BayesA, RKHS, and SVM models while with no significant difference. And the GWAS-informative SNPs was significantly superior in efficience than random selected markers by comparison of predictive performance on different selection strategies of SNPs. Overall, the genetic basis of enteritis disease resistance trait in the lined seahorse is a polygenic genetic architecture. SNPs associated with the important genes of cathepsin *L1-like* previously reported with respect to disease resistance are consider as potential molecular markers of genetic breeding. Furthermore, GS approach is an appropriate, effective, and less-cost application in breeding enteritis disease-resistant seahorses.

## Introduction

Seahorses (*Hippocampus* spp.), a group of Syngnathidae marine fish, have been one of the important ingredients in Chinese traditional medicine from time immemorial (1, 2). It is also a fascinating fish in ornamental markets for its unique body morphology. As demonstrated, at least 72 nations and territories participated in the international trading of seahorses from 1998 to 2001, and approximately 20 million seahorses were caught per year (1, 3, 4). Suffering from an unprecedented level of anthropogenic pressure promoted by habitat destruction and overexploitation (5), all 53 recognized seahorse species have been listed in CITES Appendix II of the Convention on International Trade in Endangered Species of the Wild Fauna and Flora since 2004 (1, 6). To provide a long-term solution for sustaining the seahorse trade, the commercial aquaculture of seahorses has advanced significantly in breeding and rearing protocols in the past 20 years (7–10). Particularly, the lined seahorse (*Hippocampus erectus* Perry, 1810) is considered one of the most cultivable seahorse species for its rapid growth and environment tolerance (11, 12). An unofficial source indicates that the annual cultured and dried lined seahorses have been more than 60.0 t in China since 2014 (12).

Although great progress has been achieved in the commercial culture of lined seahorses, its culture also faces serious disease challenges (13, 14). Seahorses have an anomalous immune system lacking a stomach and gut-associated lymphoid tissues and prey primarily on frozen mysis that carries abundant bacteria in *Vibrionaceae* (15, 16). These specialized morphologies and diet make seahorses particularly vulnerable to enteritis bacteria infection. Enteritis occurs primarily in juveniles with a body height of 4–6 cm and causes high die-offs of 80% within 3–5 days. The seahorses with enteritis having obvious anal openings and abdominal swelling could hardly hold the holdfast and showed weak swimming. Anatomic symptoms include liver hemorrhage, intestinal tract translucence, hindgut erosion, and ascitic fluid hoarding (14). *Vibrio*, a major bacterial pathogen, includes *V. fortis*, *V. harveyi*, *V. tubiashii*, and *V. parahaemolyticus*, which have been linked to enteritis in seahorses, including the lined seahorses (14, 17–19). The fundamental treatments, including antibiotics, provision of an optimal rearing environment, high-quality diets, periodical quarantines, and probiotics, still meet a challenge to effectively prevent or control pathogen infections in seahorse culture (8, 20, 21). To date, the immune defense mechanism in *Vibrio*-induced enteritis in seahorses is limited to physiological responses and the expression profile of some immune-related genes (e.g., *toll-like receptors 2* and *5*) (14, 22, 23, 85).

It has been widely demonstrated that disease resistance involves quantitative characteristics with a complex genetic basis and a complex trait model (24). Depending on the genome-wide association study (GWAS), a powerful and efficient method, it is possible to expound the polygenic genetic architecture from disease resistance-related loci and genes by utilizing tens of SNP markers associated with phenotypes from populations (25, 26). The whole genome-covered SNP genetic markers allow genomic selection (GS) to have overwhelming superiority in predictive accuracy and genetic gains and to facilitate genetic breeding progress (26–29). Significant genetic variation in bacterial or viral resistance has been demonstrated by GWAS in fish species, e.g., salmonids (30), olive flounder (*Paralichthys olivaceus*) (31), large yellow croaker (*Larimichthys crocea*) (28), and catfish (32, 33). Moreover, significant resistance-related candidate genes have been identified. For example, three GWAS revealed three significant SNPs associated with *plcg1*, *epha4*, *clstn2*, etc. for viral hemorrhagic septicemia virus (VHSV) resistance in olive flounder (31). *casp8* and *traf6* for *C. irritans* resistance in large yellow croaker were reported (28). Substantial evidence indicates that disease resistance has made a significant genetic improvement in aquaculture, even with lower heritability (29, 34). Considering the complex genetic basis of disease defense traits in fish, mapping resistance-related candidate genes from genomic information by GWAS is proposed as a possible and efficient strategy to have a thorough understanding of the genetic background and breeding program for enteritis disease resistance in lined seahorses.

In the present study, we challenge lined seahorses with the etiological *Vibrio harveyi* to perform a GWAS analysis and to identify the potential SNPs and candidate genes involved in genetic variation in enteritis resistance. Moreover, the potentiality of GS for enteritis resistance-related genetic improvement in seahorse farming was evaluated, by comparing the accuracies of genomic prediction of different GS models and SNP selection strategies. Thus, our study aims to have a comprehensive understanding of candidate genes in the precise localization of casual loci from a genome perspective. The present work is tremendously important for revealing the genetic mechanism and speeding up the genetic improvement for the enteritis disease resistance trait in the seahorse aquaculture industry.

## Materials and methods

### Seahorse husbandry

In this experiment, a total of 360 cultivated individuals of lined seahorses (body height 7.60 ± 0.72 cm; wet weight 1.60 ± 0.39 g) were used, which originated from a population-based cultivated seahorses from Qionghai Research Center of the East China Sea Fisheries Research Institute, Hainan, China. The seahorses were bred in a flow-through fiberglass tank (4 × 2 × 1 m) with a transparent plastic tube provided as holdfast. The seawater was sand-filtrated and ultraviolet-sterilized, maintained at a salinity of 32‰, temperature of 23–25°C, light intensity of 800–1,000 lux, and a natural photoperiod of 13 h light:11 h dark, respectively. The seahorses were fed twice a day with frozen mysis (*Neomysis awatschensis*) purchased from a harvester/supplier in Wudi County (Shandong Province, China). After mysis feeding for 1 h, the residual feeds were immediately removed to maintain the water quality. Half of the total water was changed daily. The experimental seahorses were randomly collected and transferred into 500 L plastic tanks (30 seahorses per tank) for a 3-day acclimation. The methods and experimental protocols of the present study adhered to the relevant guidelines and regulations of the Chinese Academy of Sciences under ethical approval committee no. IACUC #160413.

### *Vibrio harveyi* challenge and phenotype collection

**Figure 1:**
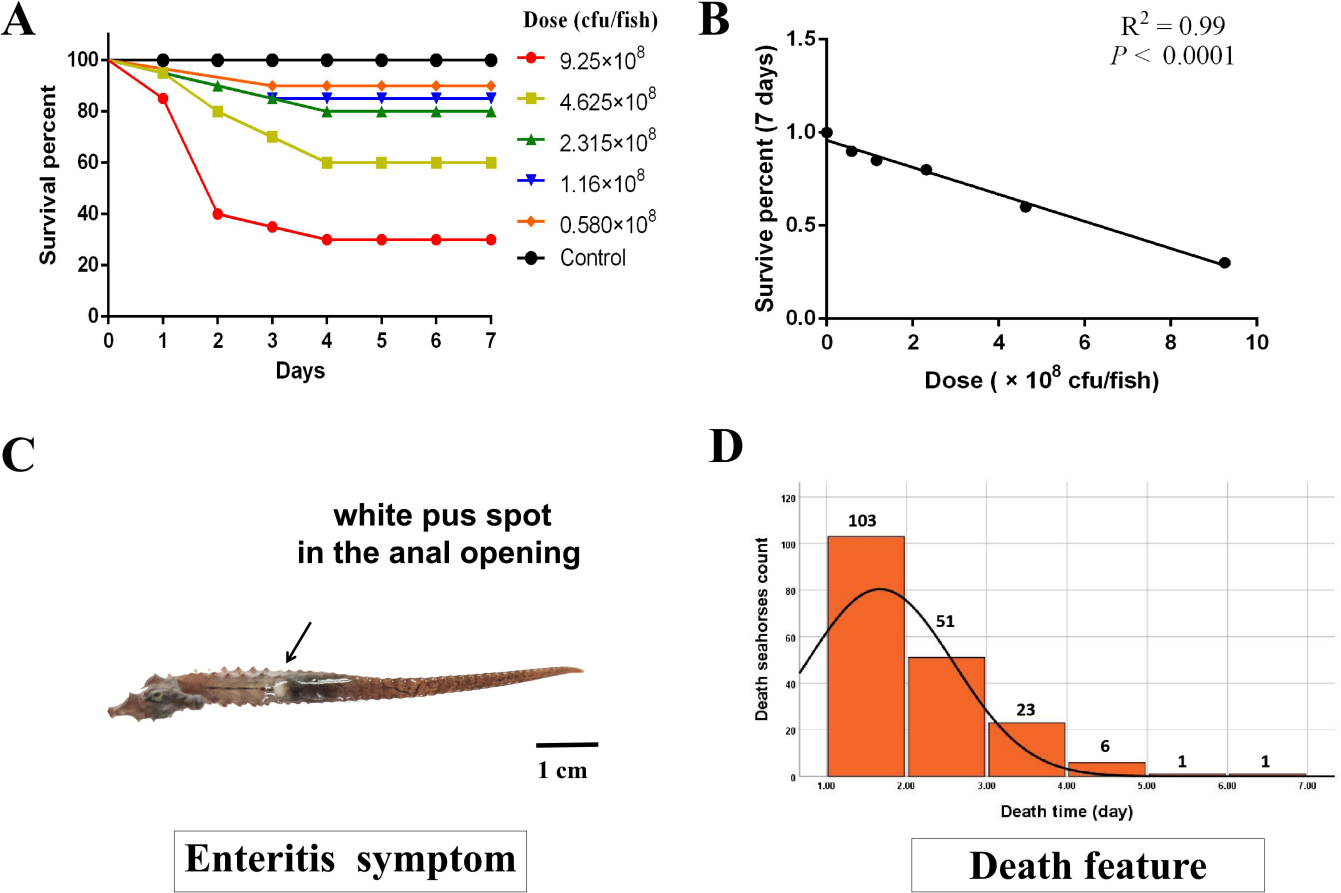
The LD_50_ of the *Vibrio harveyi* test and the corresponding death feature in lined seahorses. **(A)** The survival percentage at different *V. harveyi* infection doses. **(B)** The significant linear correlation between survival percentage and injection dose (*P* < 0.0001, goodness-of-fit test). **(C)** The typical symptom of enteritis in lined seahorses with white pus spot in the anal opening. **(D)** After injection, a mortality peak, with 103 deaths, was observed on the first day, and 51, 23, and 6 deaths were observed on the following days. On days 5 and 6, the mortality curve showed a plateau with one death, respectively. The black line denotes the density of dead individuals with the recorded time, which corresponds to the left axis.

The *V. harveyi* strain isolated from the premortal-enteritis individuals of the lined seahorse was morphologically and genetically identified according to the conventional protocol described by Lin et al. (14). The strain was prepared by culturing the bacterial isolate on thiosulfate citrate bile salts sucrose (TCBS) solution at 37°C with constant shaking (250 rpm for 24 h). The bacterial count was measured by standard dilution and by the plating method. A total of 360 experimental seahorses were then intraperitoneally injected with 50 μL of freshly cultured *V. harveyi* suspension, to a concentration of LD_50_ at 6.32 × 10^8^ cfu/fish according to the pilot study (**Figures 1A, B**). Post-injected seahorses were monitored for mortality three times a day (7:00, 12:00, and 17:00) lasting 10 days (from 15 to 25 March 2023). Dead fish was immediately removed, with the phenotypes being recorded (e.g., wet weight, body length, gender, death point, and pathological symptoms), and then stored at −80°C. On day 10, the experiment was stopped and all live seahorses were euthanized with 0.035% MS-222 (Sigma-Aldrich, Australia), the phenotypes were recorded as mentioned above, and then the tail tip tissue was sampled. The tail tips of both the dead and live individuals were stored in absolute ethanol for DNA extractions at −80°C for further genomic analysis. Resistance to *V. harveyi* infection was analyzed and defined as binary survival phenotype (status): 0 was considered susceptible once the fish died and 1 was considered resistant if the fish survived until the end of the observation period.

### Sequencing, genotyping, and quality control

Genomic DNA from 161 susceptible and 166 resistant individuals was extracted with a commercial DNA extraction kit (CWBIO, China) (33 samples failed), following the manufacturer’s instructions. Subsequently, high-quality genomic DNA was randomly fragmented by Covaris Technology, and the fragment of 350 bp was obtained after fragment selection. After PCR amplification, the single-stranded circular DNA (sscircDNA) was formatted as the final library and qualified by quality control. The qualified libraries were sequenced on the DNBSEQ-T7 platform (BGI, Shenzhen, China) at the Compass Agritechnology Co., Ltd. (Beijing, China). Then, the raw reads were obtained and filtered using SOAPnuke v2.0 by removing reads containing adapters, reads possessing N bases (ambiguous bases) >10% of the total read length, and reads with a low Phred score (Q < 5) (bases >50% of the total read length). After filtering, the clean reads of each sample were aligned against the tiger tail seahorse (*Hippocampus comes*) reference genome (GCF_001891065.2) by Burrows-Wheeler Aligner v0.7.17 with default settings (35). The SNPs and indels were further filtered using GATK v4.1.8 with the standard criteria (36): SNPs were filtered with quality by depth (QD) <2.0, Fisher strand (FS) >60.0, mapping quality (MQ) <40.0, StrandOddsRatio (SOR) >3.0, MQRankSum <−12.5, and ReadPosRankSum <−8.0; and indels were filtered with QD <2.0, FS >200.0, SOR >10.0, and ReadPosRankSum <−20.0. Indels were annotated using ANNOVAR and retained for subsequent analysis.

### Population structure and linkage disequilibrium analysis

To investigate the genetic structure of the seahorses, principal component analysis (PCA) was calculated and visualized by Plink v1.9 (37) and ggplot2 package, respectively. Furthermore, the visualization of the population structure was given by the Admixture v1.3.0 software (38) using the maximum likelihood model. Finally, genetic relatedness between individuals was evaluated using the GCTA v1.93.2 software (39), and the corresponding heatmap was visualized by the hist function in R. The linkage disequilibrium (LD) decay in susceptible and resistant populations was investigated by calculating the non-random associations among alleles at two or more loci in the PopLDdecay v3.41 package with the parameter of “-MaxDist 300 kb” (26), and the patterns of LD decay with distance were plotted using ggplot2 v3.3.6 package.

### Genome-wide association study

Based on the SNP genotype and binary survival phenotype data, the mixed linear model (MLM) of GEMMA v0.98.1 (27, 28, 40) was adopted to execute univariate GWAS analysis to detect SNPs associated with enteritis disease resistance in lined seahorses. The GWAS model is as follows: *y* = *Wα* + *Xβ* + *Zμ* + *ϵ*, where *y* is the phenotypic value (0 = susceptible, and 1 = resistant), *W* is a covariate matrix of fixed effects, *α* is the vector of the corresponding coefficients including the first three principal components, *X* is the matrix for the fixed effects, *β* is the allele substitution effect of each SNP, *Z* is the genomic kinship matrix based on SNPs, *μ* is the additive genetic effect, and *ϵ* is the vector of residual errors. The results of the univariate GWAS analysis were visualized by the “CMplot v4.1.0” package (41) in the R platform. LD pruning was carried out using Plink v1.9 (37) with the parameter of “-indeppairwise 1000kb 1 0.2” and generating independent SNPs. Based on Bonferroni correction with the estimated number of independent SNPs (*N*), the genome-wide significance association threshold was set as 0.05/*N*, and the suggestive association threshold was set as 1/*N* (26). Moreover, the significant SNPs associated with enteritis disease resistance trait in seahorses were annotated using ANNOVAR. When a significant SNP is located in or adjacent to a gene region, the gene is considered a candidate gene associated with enteritis disease resistance. To further elucidate the regulation mechanism, all candidate genes were submitted to the DAVID (https://david.ncifcrf.gov/) online website to conduct Gene Ontology (GO) enrichment and Kyoto Encyclopedia of Genes and Genomes (KEGG) pathway analysis. Finally, significantly enriched GO and KEGG terms (*P* < 0.05) were visualized with the ggplot2 v3.3.6 package.

### Genomic prediction

To evaluate the feasibility of GS in predicting enteritis resistance in the lined seahorses, the accuracies were assessed by genomic prediction (GP). Five GS prediction models, namely, conventional models of ridge regression best linear unbiased prediction (rrBLUP) (42), BayesA, and BayesC (43), and machine learning models of RKHS and SVM, were measured and compared. For conventional models, the general form of these models is as follows: *y* = *Xb* + *Zg* + *e*, where *y* is the vector of phenotypic values (0 = susceptible and 1 = resistant), *b* is the vector of fixed effect including the first three principal components, *g* is the vector of additive genetic values (SNP effect), *e* is the vector of residual effect, and *X* and *Z* are the incidence matrices relating the fixed effect and additive genetic values, respectively. Furthermore, the rrBLUP model was performed using the R package “rrBLUP” (44), and Bayes models were operated using the R package “BGLR v1.1.0” (45). In addition, two machine learning models were both kernel-based algorithms. Of these, RKHS is a semiparametric model that replaces the genomic relationship matrix with the general kernel matrix and is operated using the R package “BGLR v1.1.0” (46). SVM was implemented in the R package kernlab v0.9-32, which was originally developed as a classifier and aimed to solve the separation problems of the hyperplane with the largest geometric interval that can correctly divide a given training dataset (47). Heritability was assessed in GEMMA v0.98.1 with the following formula: *h* ^2^ = *σ* ^2^_g_/(*σ* ^2^_g_ + *σ* ^2^_e_), and *σ* ^2^_g_ and *σ* ^2^_e_, respectively.

Generally, the tagging SNPs representing SNPs in a haplotype region of the genome were adopted by Plink v1.9 (37) and used for analysis. Predictive accuracies of different GS models in enteritis disease resistance were compared by randomly selecting different numbers (10, 50, 100, 500, 1,000, 3,000, 5,000, 8,000, 10,000, and all) of SNPs using 10-fold cross-validation with 10 replicates (48). In this training set procedure, 90% of individuals were used to build the GS model and to calculate the markers effect. Meanwhile, the remaining 10% of individuals were used to calculate the genomic estimated breeding values (GEBVs). For the binary phenotype, the area under the curve (AUC) was selected as an index to evaluate prediction accuracy, and the model possessing the highest accuracy is considered as the suitable GS model for subsequent analyses.

To evaluate the possibility of considerable predictive accuracy by using a few markers, we built nine different SNP sets with the number of SNPs of 10, 50, 100, 500, 1,000, 3,000, 5,000, 8,000, and 10,000 and compared their predictive accuracies using the suitable GS model employing two SNP selection strategies as follows: selecting the SNPs with the highest ranked *P*-value based on the result of the GWAS implemented only in the training population (GWAS) and randomly selecting SNPs with five replicates (Random).

## Results

### Seahorse mortality

The concentration of LD_50_ of *V. harveyi* injection caused 54.39% of accumulated mortality (185 individuals) in 10 days, with typical enteritis symptoms of white pus spot in the anal opening (**Figure 1C**). The mortality peak, with 55.68% deaths (103 individuals), was observed on day 1 post-infection. There were 51, 23, and 6 deaths observed in the following days. On day 5, the mortality curve showed a plateau (**Figure 1D**). Considering the rapid progressive death of seahorses may not be able to separate the different resistance levels; thus, the binary survival phenotype was selected for further GWAS analysis. In general, a total of 161 and 166 susceptible and resistant individuals, respectively, were genotyped with the phenotypic statistics presented in **Table 1**. For susceptible and resistant populations, the average body height was 7.59 (SD = 0.84) and 7.62 (SD = 0.63) cm, and body weight was 1.42 (SD = 0.27) and 1.80 (SD = 0.41) g, respectively. The body weight trait ranged considerably among the experimental seahorses, with a minimum of 0.72 and 0.94 and a maximum of 2.3 and 3.15 g, respectively.

**Table 1:** Phenotypic statistics for susceptible and resistant lined seahorse populations.

Populations	Traits	Mean	SD	Min	Max
Susceptible (*N* = 161)	TH (cm)	7.59	0.84	5.2	9.5
	BW (g)	1.42	0.27	0.72	2.3
Resistant (*N* = 166)	TL (cm)	7.62	0.63	6.2	10
	BW (g)	1.8	0.41	0.94	3.15

BW, body weight; TH, total height; SD, standard deviation; Min, minimum; Max, maximum.

### Genotyping results and marker distribution

Whole-genome resequencing yielded a total of 4,677.06 Gb of raw data and 4,637.13 Gb of clean data by removing the low-quality reads. These clean data were successfully mapped to the reference genome as 89.03% of the mapped ratio. Furthermore, the average sequencing depth ranged from 14.34× to 53.23×, with an average value of 26.26× (**Supplementary Table S1**). In general, a total of 3,816,855,962 SNPs passed quality control and the transition to transversion ratio is 1.30. Of these identified SNPs, 26.20% were distributed in the intergenic region, 6.43% were in the exon region, 56.11% were in the intron region, and 6.32% were in the 1-kb region up- and downstream of the gene coding sequences (**Supplementary Table S2**), respectively. Adopting genomic information, the heritability was estimated as ~0.10 for enteritis resistance of the lined seahorses.

### Population structure and linkage analysis

**Figure 2:**
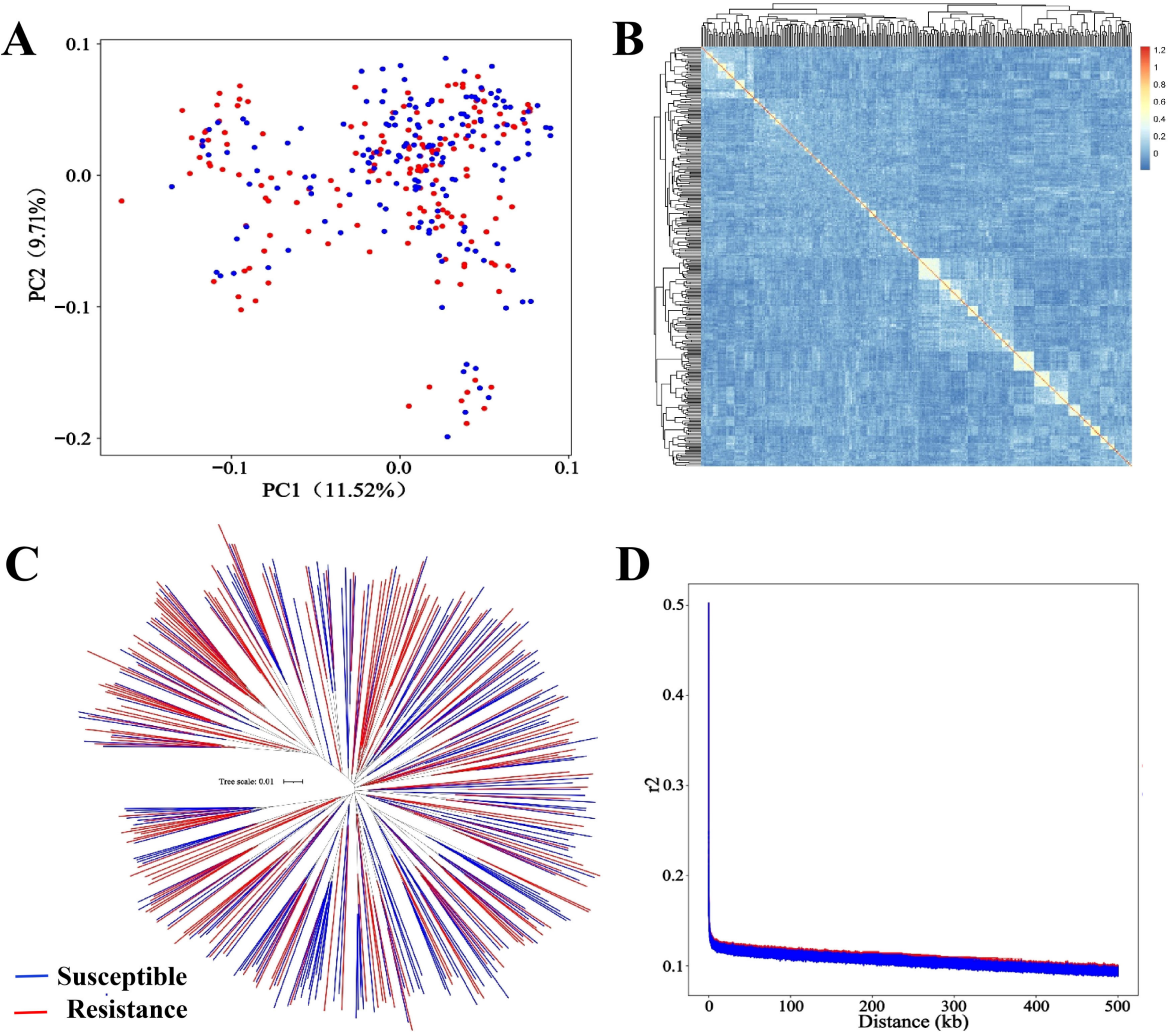
Visualization of the population structure in the challenged lined seahorses. **(A)** Population structure indicated by PCA with the first two principal component scores. **(B)** The heatmap of genetic relatedness. **(C)** Population phylogeny analysis. **(D)** Genome-wide linkage disequilibrium (LD) decay plots of SNPs for susceptible and resistant lined seahorses under *V. harveyi* infection for 10 days. Susceptible and resistant individuals are presented as red and blue, respectively.

According to the PCA results, the first two principal components explained 11.52% and 9.71% variance of the genetic structure, respectively. Susceptible and resistant lined seahorses belong to the same genetic group as they share an overlapped cluster (**Figure 2A**). Furthermore, the genetic relatedness reveals no/a slight genetic relatedness among susceptible and resistant individuals (**Figure 2B**). No population stratification was identified, which was further verified by population structure analysis (**Figure 2C**). Linkage disequilibrium across the markers was plotted using genotype data presenting the LD decay with increasing marker distance (**Figure 2D**). The squared correlation coefficient (*r* ^2^) of two loci decreased dramatically increasing the distance of each pair of SNPs, and the *r* ^2^ value was 0.1 when the distance was approximately 300 kb.

### GWAS analysis results and candidate gene identification

**Figure 3:**
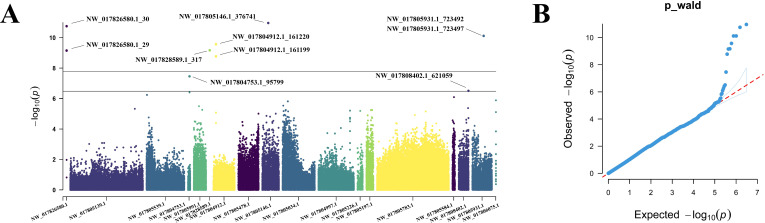
Manhattan plot **(A)** and quantile–quantile (Q-Q) plot **(B)** of enteritis resistance trait in GWAS analysis for the lined seahorse population. In the Manhattan plot, the upper gray line and the lower black line present the threshold *P*-value for genome-wide significance and suggestive significance, respectively. The significant SNPs were specified.

**Figure 4:**
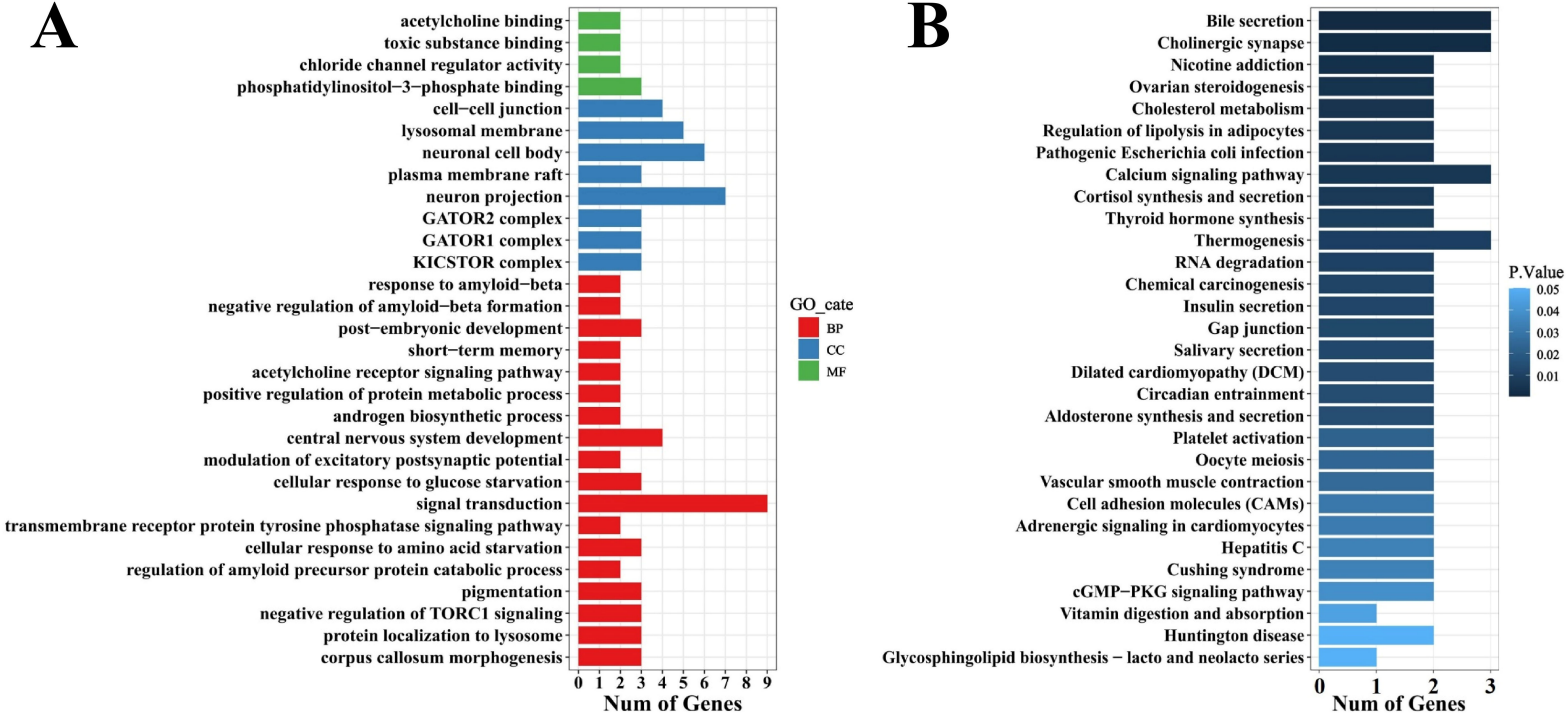
Gene ontology (GO) **(A)** and KEGG **(B)** enrichment analysis of candidate genes associated with enteritis disease resistance in lined seahorses. The top 30 items are presented.

Univariate GWAS analysis was performed and eight genome-wide significant and two suggestive SNPs were identified to be associated with enteritis resistance in lined seahorses, mainly on seven chromosomes, as visualized in the Manhattan and Q–Q plots in **Figure 3**. The summary of the significant SNPs associated with the enteritis resistance trait is shown in **Table 2**. The minimum allele frequency (MAF) of these SNPs ranged from 0.063 to 0.417, and the phenotypic variance explained (PVE) ranged from 7.76% to 13.28%. Correspondingly, 63 genes that exceeded the significant threshold and 19 genes that passed the suggestive threshold were identified as putative candidate genes associated with enteritis resistance in lined seahorses (**Supplementary Table S3**). Flanking the most important SNP of scaffold NW_017805146.1_376741, nearly one-third of the candidate genes were found. These harbored candidate genes were classified into 419 significant GO terms (**Figure 4A**, detailed information is shown in **Supplementary Table S4**) and 29 significant KEGG pathways (**Figure 4B**, detailed information is shown in **Supplementary Table S5**), involved in fundamental functions in biological processes.

**Table 2:** Summary of the significant SNPs associated with enteritis disease resistance in GWAS analysis for the lined seahorse population.

SNP ID	Position (bp)	Allele	Location	MAF	PVE	*P*_wald
NW_017805146.1_376741	376,741	A/G	Intronic	0.07	0.13	1.09045E−11
NW_017826580.1_30	30	G/A	Intergenic	0.11	0.13	1.75885E−11
NW_017805931.1_723492	723,492	G/C	Intergenic	0.07	0.12	7.62848E−11
NW_017805931.1_723497	723,497	A/T	Intergenic	0.07	0.12	7.62848E−11
NW_017804912.1_161199	161,199	T/C	Intronic	0.06	0.11	1.6782E−09
NW_017804912.1_161220	161,220	C/T	Intronic	0.08	0.12	2.69682E−10
NW_017828589.1_317	317	G/A	Intergenic	0.07	0.11	6.7707E−10
NW_017826580.1_29	29	C/G	Intergenic	0.10	0.11	7.00878E−10
NW_017804753.1_95799*	95,799	C/G	Exon	0.08	0.09	3.48262E−08
NW_017808402.1_621059*	621,059	C/A	Intronic	0.42	0.08	3.10693E−07

* indicates suggestive significant level; others are at the genome-wide significant level.

MAF, minimum allele frequency; PVE, phenotypic variance explained.

Particularly, as shown in **Table 3**, positive regulation of epidermal cell differentiation (GO:0045606), actin filament organization (GO:0007015), endothelial cell morphogenesis (GO:0001886), and positive regulation of cell population proliferation (GO:0008284) signal pathways were highly clustered. We also observed that cellular response to starvation (GO:0009267), cellular response to epinephrine stimulus (GO:0071872), and response to hypoxia (GO:0001666) related to the early response of external stress were significantly enriched in the present study. Candidate genes (*death-associated protein kinase 2-like*, *wdr45*, *cathepsin L1-like*, *sorting nexin-27-like*, *protein SZT2-like*, *CHRNA7-FAM7A fusion protein-like*, *adenylate cyclase type 2-like*, and *neuronal acetylcholine receptor subunit alpha-7-like*) grouped into immune-related pathways, such as autophagy (GO:0000422; GO:0006914), protein localization to lysosome (GO:0061462), leukocyte migration involved in inflammatory response (GO:0002523), and toll-like receptor 4 signaling pathway (GO:0034142), also exhibited enrichment and were annotated as a response to bacterial infection in lined seahorses.

**Table 3:** GO terms related to enteritis disease resistance trait in lined seahorse.

GO_term	ID	*P*-value	Gene name
Disease defense			
Protein localization to lysosome	GO:0061462	0.0000	*protein SZT2-like*
Cellular response to muramyl dipeptide	GO:0071225	0.0182	*arhgef2*
Negative regulation of tumor necrosis factor production	GO:0032720	0.0039	*CHRNA7-FAM7A fusion protein-like; neuronal acetylcholine receptor subunit alpha-7-like*
Leukocyte migration involved in inflammatory response	GO:0002523	0.0218	*fut7*
Negative regulation of platelet aggregation	GO:0090331	0.0236	*cGMP-dependent protein kinase 1-like isoform X1*
Negative regulation of necroptotic process	GO:0060546	0.0271	*arhgef2*
Toll-like receptor 4 signaling pathway	GO:0034142	0.0342	*s100a14*
Autophagy of mitochondrion	GO:0000422	0.0025	*wdr45; cathepsin L1-like*
Autophagy	GO:0006914	0.0232	*wdr45; lix1l*
Positive regulation of apoptotic process	GO:0043065	0.0243	*death-associated protein kinase 2-like isoform X1; arhgef2; triple functional domain protein-like isoform X1*
Epidermal cell differentiation			
Positive regulation of epidermal cell differentiation	GO:0045606	0.0164	*keratinocyte differentiation factor 1-like*
Positive regulation of cell population proliferation	GO:0008284	0.0139	*CHRNA7-FAM7A fusion protein-like; kdm4a; brachyury protein homolog A; neuronal acetylcholine receptor subunit alpha-7-like*
Actin filament organization	GO:0007015	0.0205	*arhgef2; tropomyosin alpha-3 chain*
Positive regulation of leukocyte adhesion to vascular endothelial cell	GO:1904996	0.0234	*fut7*
Myofibril assembly	GO:0030239	0.0253	*klhl41*
Intestinal absorption	GO:0050892	0.0271	*scarb1*
Endothelial cell morphogenesis	GO:0001886	0.0271	*chloride intracellular channel protein 4-like*
Positive regulation of cell–cell adhesion	GO:0022409	0.0342	*fut7*
Epigenetic regulation			
Negative regulation of histone H3-K9 trimethylation	GO:1900113	0.0109	*kdm4a*
Histone H3-K36 demethylation	GO:0070544	0.0200	*kdm4a*
Histone H3-K9 demethylation	GO:0033169	0.0289	*kdm4a*
Regulation of DNA methylation	GO:0044030	0.0253	*grainyhead-like protein 2 homolog isoform X1*
Response to stress			
Response to hypoxia	GO:0001666	0.0357	*CHRNA7-FAM7A fusion protein-like; neuronal acetylcholine receptor subunit alpha-7-like*
Response to fungicide	GO:0060992	0.0127	*srd5a1*
Cellular response to starvation	GO:0009267	0.0082	*srd5a1; wdr45*
Cellular response to epinephrine stimulus	GO:0071872	0.0253	*srd5a1*
Cellular response to interleukin-7	GO:0098761	0.0253	*ATP synthase subunit beta, mitochondrial*
Neuroendocrine			
Signal transduction	GO:0007165	0.0001	*arhgap39; sorting nexin-27-like; neuronal acetylcholine receptor subunit alpha-7-like; CHRNA7-FAM7A fusion protein-like; artn; brachyury protein homolog A; cGMP-dependent protein kinase 1-like isoform X1; kifap3; 14-3-3 protein zeta-like isoform X1*
Modulation of excitatory postsynaptic potential	GO:0098815	0.0001	*CHRNA7-FAM7A fusion protein-like; neuronal acetylcholine receptor subunit alpha-7-like*
Central nervous system development	GO:0007417	0.0001	*protein SZT2-like; triple functional domain protein-like isoform X1*
Androgen biosynthetic process	GO:0006702	0.0002	*srd5a1; scarb1*
Acetylcholine receptor signaling pathway	GO:0095500	0.0003	*CHRNA7-FAM7A fusion protein-like; neuronal acetylcholine receptor subunit alpha-7-like*
cAMP biosynthetic process	GO:0006171	0.0200	*adenylate cyclase type 2-like*
Regulation of insulin receptor signaling pathway	GO:0046626	0.0253	*fut7*
Thyroid hormone generation	GO:0006590	0.0325	*cathepsin L1-like*
Androgen receptor signaling pathway	GO:0030521	0.0360	*protein TMEPAI-like*
cGMP-mediated signaling	GO:0019934	0.0395	*cGMP-dependent protein kinase 1-like isoform X1*

Interestingly, in **Figure 4**, we found the importance of regulation of the neuroendocrine on immune response, as the majority of the KEGG pathways were enriched in the neuroendocrine network associated with the resistance of the lined seahorses against *V. harveyi*, e.g., the neurohormonal system, including cortisol synthesis and secretion (hsa04927), thyroid hormone synthesis (hsa04918), insulin secretion (hsa04911), and ovarian steroidogenesis (hsa04913). Notably, related genes (*scarb1*, *adenylate cyclase type 2*, *lrp2*, and *cGMP-dependent protein kinase 1-like*) that participated in digestive pathways (e.g., bile secretion, cholesterol metabolism, salivary secretion, and vitamin digestion and absorption) were heavily enriched and demonstrated its fundamental function in bacterial resistance in lined seahorses. Among the candidate genes, *adenylate cyclase type 2-like* and *scarb1* had a high frequency, participating in multiple signal pathways in response to infection. These genes are highly related to the biological processes of adenylated cyclase activity and scavenger receptor activity, assuming a fundamental role in enteritis resistance in lined seahorses.

### Genomic prediction

**Figure 5:**
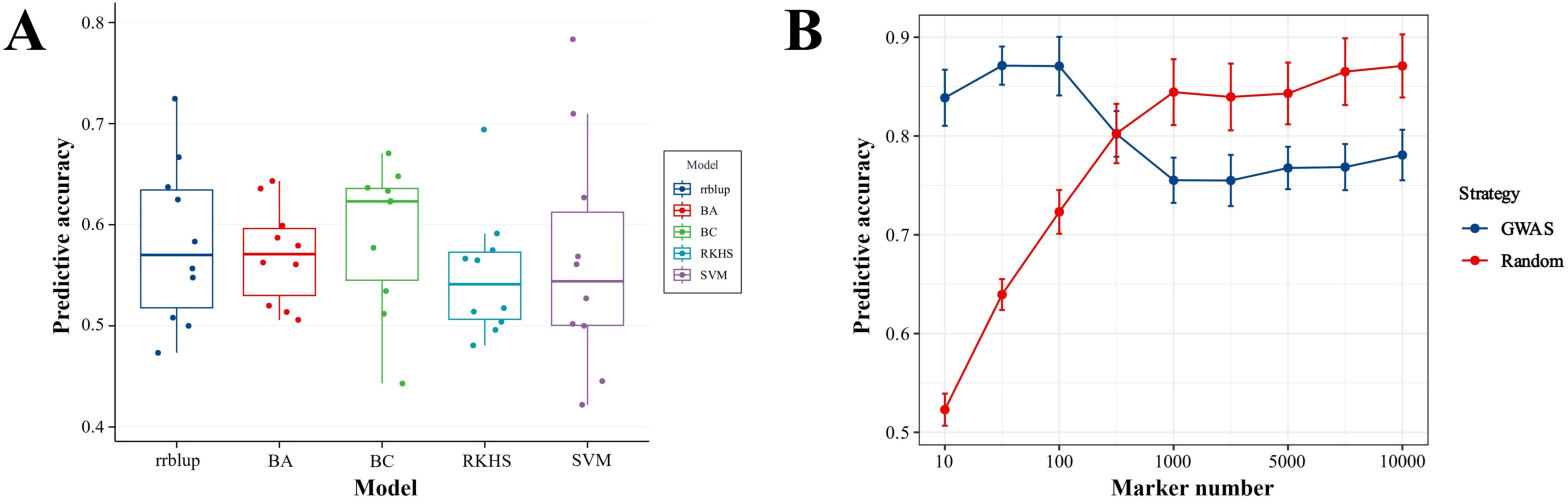
Comparison of predictive accuracies under different models **(A)** and selection strategies **(B)** for enteritis disease resistance in lined seahorses. **(A)** No significant difference in predictive accuracies was observed between the three conventional models (rrBLUP, BA, and BC) and the two machine learning models (RKHS and SVM) (*P* > 0.05, one-way ANOVA). **(B)** The predictive accuracies of GWAS and Random selection strategy under different numbers of SNP sites.

A total of 319,099 tagging SNPs were retained after Plink filtration and were applied to estimate the predictive performance of three conventional models (rrBLUP, BA, and BC) and two machine learning models (RKHS and SVM) through randomly adopting different numbers of SNPs of a 10-fold cross-validation analysis. As shown in **Figure 5A**, the predictive accuracy of BayesC reached 0.59, higher than those of rrBLUP (0.58), BayesA (0.57), RKHS (0.55), and SVM (0.58) with no significance (*P* > 0.05, one-way ANOVA). This result indicated that BayesC was considered as the suitable model and was selected for further GWAS analysis. Furthermore, the influence of different SNP selection strategies and different marker densities on prediction accuracy was assessed by comparison of the GWAS and Random selection strategies under different numbers of SNP sites. As demonstrated in **Figure 5B**, the predictive accuracy of the GWAS strategy was 0.871 of the predictive plateau under 100 of marker density, which is higher than the Random selection strategy of 0.723. When under 500 of marker density, the predictive accuracy of GWAS and Random was 0.802 and 0.803, respectively. On the contrary, when the marker density was increased above 1,000, the predictive accuracy of the GWAS strategy was lower than that of the Random strategy, being 0.755–0.781 and 0.840–0.871, respectively. Overall, these results implied that the GWAS strategy can achieve better prediction accuracy at a lower marker density.

## Discussion

*Vibrio harveyi* is a major bacterial pathogen causing enteritis disease in seahorses (18). In the present study, the concentration of LD_50_ of *V. harveyi* causes 54.39% of accumulated mortality, with typical enteritis symptoms of white pus spot in the anal opening as reported before (14). In infected seahorses, a death rate of 95.68% mainly occurred within the first 3 days, unlike the death rate of rainbow trout with a bacterial cold-water infection lasting 21 days (49). Previous studies have shown that a certain concentration of bacteria had a stimulating effect on fish (28). The highly enriched stress-related KEGG pathways in the present study further inferred that the stimulating effect induced by *V. harveyi* may be responsible for the short-time duration of death in lined seahorses. Furthermore, this short-time duration made it insufficient to distinguish the difference in resistance among individuals; thus, binary survival was adopted in our GWAS analysis as phenotype. As reported, the binary survival phenotype was considered to better present disease resistance than survival time as the latter fit more the conception of disease endurance or tolerance measurement (28, 50). In some studies, the genomic heritability for binary survival status is greater than survival days after challenge. As exemplified, values of 0.59 and 0.38 in *Cryptocaryon irritans* resistance in large yellow croaker (28) and 0.27 and 0.14 in *Piscirickettsia salmonis* resistance in coho salmon (30) were reported for binary and quantitative traits, respectively. However, this is not always the case. The heritability values for *P. salmonis* resistance in rainbow trout were 0.48 using day of death and 0.34 for binary survival as trait definitions (51). Different model applications may be an important point of consideration related to these outcomes (28).

No population stratification and a rapid LD decay in susceptibility and resistance of the lined seahorses are identified, which further ensures the reliability of GWAS analysis in the present study (52, 53). Generally, a set of 10 enteritis resistance-related SNPs with PVE ranging from 7.76% to 13.28% is identified, and there is extensive distribution across seven chromosomes by GWAS analysis using the mixed linear model. Our case study supports a proposal that enteritis disease resistance in lined seahorses is a polygenic genetic architecture trait, regulated by many micro-effect genes rather than some major QTLs (54, 55). This result is consistent with disease resistance in large yellow croaker (1.06%–7.26% with 15 QTLs) (28) and rainbow trout (9% with one SNP) (56). Though not always, two significant QTLs with 83% genetic variance were identified in the Atlantic salmon (*Salmon salar*) population against infectious pancreatic necrosis (IPN) (57, 86). As illustrated, the genetic architecture of disease resistance trait is highly dependent on the given disease and fish species. Furthermore, the heritability for resistance to *V. harveyi* in lined seahorses is ~0.10, moderately lower than those within the range of estimations in aquaculture species, ranging from 0.16 to 0.27 of heritability in coho salmon (*Oncorhynchus kisutch*) (30, 58). Nevertheless, when increasing sample numbers to 2,407 fish with a higher number of generations of artificial selection, heritability reached a moderately higher value of 0.34 for the binary survival for *P. salmonis* resistance in rainbow trout (51). Despite the moderately low heritability for resistance against *V. harveyi* obtained in the present study, significant SNPs further suggest the effectiveness of selection for resistance improvement.

In addition, only one of these SNPs was located in the exon region, causing homozygous mutation, while other SNPs were found located in intronic and intergenic regions. These results imply that genetic variation-related resistance trait may be through regulation rather than changing gene products directly (59). Interestingly, we indeed find three candidate genes related to epigenetic modification, namely, *methyltransferase-like protein 5* (*mettl5*), involved in rRNA methylation (GO:0031167); *lysine-specific demethylase 4A* (*kdm4a*), involved in histone H3-K9 demethylation (GO:0033169), regulation of DNA methylation (GO:0044030), and negative regulation of histone H3-K9 trimethylation (GO:1900113); and *THAP domain-containing protein 7* (*thap7*), involved in negative regulation of histone acetylation (GO:0035067). Epigenetic modification is suggested to be one of the most important molecular mechanisms involved in plastic adaptive response to environmental changes. Ma et al. (60) demonstrated that DNA methylation regulates the expression of immune-related genes involved in the PI3K–Akt signaling pathway, which enables *P. olivaceus* to adapt to adverse environmental stresses by resisting apoptosis. Li et al. (61) had proved insightful information on epigenetic regulation of immunity-related genes in *Crassostrea gigas* upon *Vibrio alginolyticus* infection by whole-genome DNA methylation profiling.

The SNP that explained the greatest proportion of genetic variance in the present study is located on the scaffold of NW_017805146.1_376741, in a region that contains a gene that encodes *grainyhead-like protein 2* (*Grhl2*). Grhl2, a transcription factor, was clustered into cell junction assembly and regulation of keratinocyte differentiation signaling pathways. As reported, this protein regulates the expression of cell junction proteins such as E-cadherin and vimentin that are key to epithelial morphogenesis and barrier formation process (62, 63). Meanwhile, *keratinocyte differentiation factor 1-like*, involved in positive regulation of the epidermal cell differentiation signal pathway, was proved to be a significant candidate gene. Previous studies have shown that enteritis symptoms are of intestinal tract translucence and hindgut erosion in lined seahorses (14). The highly clustered GO terms regarding cell differentiation and organization signal pathways implied that the proliferation phase followed by the acceleration of keratinocyte and fibroblast proliferation and migration is important for restoring the epidermis and dermal layers under inflammatory conditions (87, 88). Therefore, one might speculate that a significant part of the resistance machinery against enteritis disease should be the restoration of intestine integrity, as the intestine is an important immune organ of fish, serving as the first line of defense against microbial invasion (64, 65).

Resistance to infection can be the result of a host gaining a more robust immune response to the invading agent. On the scaffold of NW_017805146.1_376741, *cathepsin L1-like*, its protein is a crucial enzyme in the cysteine family and a typical lysosomal protease (66), expressed in a variety of immune tissues (67). *Cathepsin L* is suggested as one of the important molecules functioning in Toll-like receptor 9 signaling, activation of interleukin-8, regulation of β-defensin activity, and antigen presentation in the immune responses of vertebrates against pathogen invasion (68). The potential role of *cathepsin L* as a chemical barrier against microbial invasion in fish innate immune response has been widely demonstrated (69–71). As illustrated, lipopolysaccharide (LPS) or *Edwardsiella tarda* induced the expression of *cathepsin L* involved in the immune defense system of the rock bream (*Oplegnathus fasciatus*) (69). *Cathepsin S* was further identified by GWAS as an important candidate gene responding to bacteria and wounding signal terms in the large yellow croaker *C. irritans* resistance trait (28). Interestingly, our comparative transcriptome data (not published) show that eight cathepsin genes showed different expression responses among mock, susceptible, and resistant individuals. Importantly, *cathepsin L1-like* and *B* significantly elevated their expression when responding to *V. harveyi*, further indicating their immune function against bacterial infection.

In our study, three SNPs (NW_017804753.1_95799, NW_017805931.1_723492, and NW_017805931.1_7234927) were located in regions that contain the *E3 ubiquitin-protein ligase UBR3* (*ubr3*) and *scavenger receptor class B member 1* (*scarb1*) genes, respectively. The roles of *ubr3* mediating the proteasomal degradation of target proteins and *scarb1* in recognition of the apoptotic cell open the possibility that these two processes could be important factors in *V. harveyi* disease resistance in lined seahorses. The difference in ubiquitin-dependent and apoptosis processes has illustrated the importance of resistance to infection (56, 72). As reported, the candidate genes *E3ubiquitin-protein ligase HUWE1* and *Apoptosis-stimulating of p53 protein 2-like* were identified to be associated with the IPNV resistance trait in Atlantic salmon (56). *Scavenger receptor class B member 1* (*scarb1*), belonging to the subfamilies of the pattern recognition receptor (PRR) family, has been suggested to be involved in the clearance and detoxification of endotoxin as defense against invading pathogens in animals (73, 74).

Furthermore, two SNPs (NW_017804912.1_161199 and NW_017804912.1_161200) were located close to *cadherin-2-like* (*Cdh2l*), which encoded a protein with a pivotal role in tissue morphogenesis and disease (75). *Cdh1* had been identified as a causative gene and elaborated the machinery in the internalization of viruses, to determine resistance in Atlantic salmon against IPNV (76). Particularly, *rho guanine nucleotide exchange factor 2* (*arhgef2*) and *tropomyosin alpha-3 chain* (*tpm3*) and *unconventional myosin-XVIIIb-like isoform* were involved in actin filament organization in response to infection. As reported, the utilization of clathrin for internalization and, subsequently, the actin cytoskeleton for vacuole generation were reported as a bacterial defense pathway in fish and other mammals (51). These identified SNPs and candidate genes show an array of possibilities for enteritis disease resistance improvement screening in the practical breeding of seahorses. Nevertheless, the present study is far from sufficient to elaborate the complex traits of enteritis disease resistance in lined seahorses, and large and robust datasets should be generated to replenish the present results.

The extensive application of GS has been made to enhance resistance traits in aquaculture species, such as amoebic gill disease resistance in Atlantic salmon (77) and *C. irritans* and *Pseudomonas plecoglossicida* disease resistance in large yellow croaker (78, 79). It is important to adopt an appropriate GS model considering the accumulative burden of various factors, e.g., reference population size, genetic relatedness, population structure, and heritability, affecting the accuracy of genomic prediction (80, 81). In the present study, the predictive accuracy of the BayesC model was comparatively higher; however, there was no significant difference among the other models. It has been generally accepted that increasing population size can enhance predictive accuracy effectively in GS studies (82–84). As we added genetic relatedness and population structure as covariates to the model, the small number of genotyping individuals and low heritability may elucidate this result. Taking economics into consideration, it is necessary to obtain considerable predictive accuracy by using a few markers as possible. We further explored the impact of reduced SNP density on predictive accuracy of the GS model and the Random selection strategy. As demonstrated, the predictive accuracy of GWAS outperforms that of the Random strategy with a high level of 0.87 under 500 of marker density, suggesting that the GWAS strategy is feasible and efficient in genomic prediction. Therefore, in further studies, the adoption of a practical, highly efficient, and accurate GP selection strategy should consider various factors based on specific species and traits.

## Conclusion

In the case of seahorse culture, vibriosis has been the most concerning and common bacterial pathogen that causes substantial economic and production losses. To the best of our knowledge, the present work firstly elucidates the detection and position of SNPs and the candidate genes involved in enteritis disease resistance in a farmed lined seahorse population by GWAS analysis. Generally, resistance to *V. harveyi* could be described as a polygenic genetic architecture trait with ~0.10 of heritability since a set of 10 SNPs with a low effect were identified. The 82 potential candidate genes, with a biological role in fish signal transduction, cell proliferation, response to external stress, bacterial defense, and anti-inflammatory processes, suggest that they could participate in the mechanisms of seahorse resistance against enteritis disease. Moreover, BayesC is considered as the relatively accurate GS model for predicting the performance of enteritis disease resistance trait in lined seahorses. We further verified the efficiency of GWAS in achieving better prediction accuracy even at a lower marker density. Overall, the present study gains a genetic basis and mechanistic insights regarding resistance to enteritis disease in lined seahorses and aids in formulating guidelines for selective breeding programs to ensure enteritis disease resistance-related genetic improvement in seahorse farming.

## Data Availability

The datasets presented in this study can be found in online repositories. The names of the repository/repositories and accession number(s) can be found in the article/**Supplementary Material**.
